# Ventral Hernia Repair: A Journey from Laparoscopic to Robotic Surgery: Is Cost Efficiency Guaranteed?

**DOI:** 10.3390/jcm14113909

**Published:** 2025-06-02

**Authors:** Marco Milone, Pietro Anoldo, Michele Manigrasso, Anna D’Amore, Carmine Iacovazzo, Giuseppe Servillo, Giovanni Domenico De Palma

**Affiliations:** 1 Department of Clinical Medicine and Surgery, University of Naples ‘Federico II’, Via Pansini 5, 80131 Naples, Italy; milone.marco.md@gmail.com (M.M.); michele.manigrasso@unina.it (M.M.); anna.damore1993@libero.it (A.D.); giovanni.depalma@unina.it (G.D.D.P.); 2 Department of Neuroscience, Reproductive Science and Odontostomatological Science, University of Naples ‘Federico II’, Via Pansini 5, 80131 Naples, Italy; carmine.iacovazzo@unina.it (C.I.); giuseppe.servillo@unina.it (G.S.)

**Keywords:** hernia surgery, robotic surgery, l-IPOM, rTA-RM, cost analysis

## Abstract

**Background/Objectives**: Ventral hernia repair has evolved with the introduction of minimally invasive techniques like l-IPOM and rTA-RM. While robotic surgery offers advantages in precision and ergonomics, its higher costs pose questions regarding its cost-effectiveness compared to laparoscopic approaches. **Methods**: A retrospective analysis of patients with primary or incisional ventral hernias undergoing either l-IPOM or rTA-RM between February 2022 and October 2023 was conducted. Data on demographics, surgical outcomes, hospital costs, disposable supplies, and robotic system expenses were collected. A one-to-one propensity score matching (PSM) was used to ensure comparability between the groups. **Results**: After matching, 30 patients were included in each group. The rTA-RM group had longer operative times (93.2 vs. 74.4 min, *p* = 0.004) but shorter hospital stays (1 day vs. 2 days, *p* = 0.003) and lower postoperative pain scores (median VAS score 3 vs. 5, *p* = 0.004). Total costs were comparable between rTA-RM and l-IPOM (EUR 6862 vs. EUR 6575, *p* = 0.32), with robotic surgery incurring higher capital costs but lower disposable supply costs (EUR 1057 vs. EUR 2006, *p* < 0.01). **Conclusions**: Despite the higher per-case cost associated with robotic systems, overall costs for rTA-RM were similar to those for l-IPOM, suggesting that robotic surgery may be cost-competitive due to lower disposable supply expenses and shorter hospital stays. Further research is needed to assess long-term outcomes and broader economic impacts.

## 1. Introduction

Despite ventral hernias being one of the most common pathologies seen by clinicians, surveys and review of nationwide databases of patients undergoing elective ventral hernia repair demonstrate substantial heterogeneity in clinical management [1].

Among several surgical interventions, the management of primary or incisional ventral hernias stands out as a domain witnessing significant innovation, particularly with the advent of minimally invasive approaches in order to minimize postoperative complications and enhance overall outcomes, including recovery and recurrence rates [2]. Laparoscopic intraperitoneal onlay mesh (l-IPOM) and robotic transabdominal retromuscular mesh repair (rTA-RM) represent two forefront methodologies in this arena, each with its unique advantages and challenges [3,4]. Particularly, robotic repair has acquired remarkable consideration as a valid alternative to laparoscopic surgery thanks to the feasibility of suturing, the precision of endowrist instruments, and surgeon ergonomics [5]. However, one of the limitations of robotic surgery is represented by the high costs, which often do not justify the use of the robotic platform [6]. Research to optimize patient outcomes while ensuring cost-effectiveness remains critical, especially considering that there is much conflicting data in the literature regarding the cost-effectiveness of robotic ventral hernia repair [7].

This article aims to delve into a comprehensive cost analysis of these two techniques, shedding light on the economic implications that accompany their clinical outcomes. By juxtaposing l-IPOM against rTA-RM, we endeavor to provide an understanding of their relative cost-efficiency, considering not only the direct surgical expenses but also the broader economic impact related to recovery times and complication rates. Through this analysis, healthcare providers, policy makers, and patients alike can gain valuable insights into the economic dimensions of choosing the most appropriate surgical approach for ventral hernias, thereby facilitating informed decision making in the pursuit of optimal care.

## 2. Materials and Methods

### 2.1. Patient Population

This study was performed in accordance with the principles of the Declaration of Helsinki and its appendices. Ethical approval was obtained from the institutional review board, and informed consent was obtained from all individual participants included in the study. We conducted a retrospective analysis from a prospectively maintained database of consecutive patients affected by primary or incisional ventral hernia who underwent l-IPOM and rTA-RM at a single center between February 2022 and October 2023. The procedures were performed by a surgeon who had expertise in laparoscopic surgery and robotic-assisted surgery for abdominal wall hernias and served as a robotic abdominal wall hernia proctor for the da Vinci^®^ Surgical System(Surgical Intuitive, Inc., Mountain View, CA). Our experience consisted of the treatment of the aforementioned hernias with the laparoscopic IPOM technique, then moving on to the robotic technique. The first 2 robotic cases were performed with the IPOM technique until the definitive adoption of the retromuscular approach. Robotic IPOM cases were excluded from the analysis. Patients were excluded from the study if they were younger than 18 or older than 85 years, and those undergoing ventral hernia repair concomitantly with a second major procedure were also excluded.

### 2.2. Data Collection and Outcomes

The following data were collected: patient’s demographic information (age, gender, BMI), American Society of Anesthesiologists Physical Status Classification (ASA class), comorbidities, hernia presentation (primary vs. recurrent), operative details, surgery cost measures, length of stay, 30-day readmissions and reoperations, time to return to work, and rate severity of complications according to Clavien–Dindo classification [8]. Operative details included surgical technique (rTA-RM vs. l-IPOM); operative time; type of mesh; and use of tacks, suture, or sealant to secure the mesh.

### 2.3. Cost Analyses

Cost information was provided by the institution’s financial department and compared between the 2 groups. Three separate cost analyses were obtained and are presented:

1. Total hospital costs: this included the estimated cost of anesthesia, operating room, and recovery in addition to the disposable supplies and medications used during surgery. Only costs associated with the index admission for the ventral hernia repair procedure were included. Costs of any readmission and reoperation were not included in this analysis.
2. Total disposable supplies and specific categories costs: data combining detailed operating room usage with actual supply pricing were used for this analysis. The amount and costs for trocars, fixation devices, meshes, medications, drapes, and all accessories and other disposable equipment and parts were collected. Cost was adjusted to the 2022 Euro value. Total costs of the disposable supplies were tabulated and compared between the 2 groups.
3. Cost of the Robotic da Vinci^®^ Surgical Systems: the capital cost associated with utilizing the robot per case was calculated as the total depreciation of the capital cost during the study period divided by the number of all and any robotic cases performed by all surgeons at our institution during the same time period. The cost of the maintenance services per case was also calculated, and the total added cost was noted.

### 2.4. Operative Technique

l-IPOM repair: The patient is placed supine with both arms tucked in to allow adequate space for the surgeon and the assistant on the same side of the table. A Veress needle is inserted at Palmer’s point to create pneumoperitoneum. Most case procedures are performed with lateral ports with a 10-mm camera port placed in the lumbar region in line with the umbilicus just anterior to the midaxillary line. Additional two 5-mm working ports are inserted under vision. The pressure is maintained at 12–15 mmHg. A 30-degree telescope is used for all cases. Hernial contents are reduced by applying gentle traction with a grasper and manual pressure from the outside. However, adhesiolysis with cold scissors or ultrasonic shears is needed in some cases with dense adhesions. In some cases, the falciform ligament and the bladder flap are taken down to make adequate space for mesh placement. In cases that allow it, the fascial defect is closed with non-absorbable sutures before positioning the mesh (IPOM plus). A composite mesh of an appropriate size is tailored for a minimum 5 cm overlap in all directions. The preplaced central suture on the mesh is sequentially withdrawn through the abdominal wall using a suture passer. The mesh perimeter is secured with absorbable tacks placed at 1 cm intervals after tactile perception from the abdominal wall. A second row of tacks is applied at approximately 2 cm intervals and 2 cm from the edge (double crown technique).

rTA-RM repair: The patient’s position and creation of the pneumoperitoneum are set as for the previously described technique. Three robotic ports are inserted in appropriate sites depending on hernia location, usually along the midaxillary line, spaced approximately 8 cm apart. The assistant port (AirSeal^®^ (CONMED Corp., Largo, FL, USA) access port) is placed along the same line or behind it, triangulated with robotic ports. The patient side cart of the da Vinci^®^ surgical robotic system is docked. The robotic instruments used, in addition to the 30-degree robotic endoscope, are Hot Shears, Fenestrated Bipolar Forceps, and a Large Needle Driver. Adhesiolysis is performed as needed. The posterior rectus fascia is then cut, and retrorectus dissection is performed according to the hernia defect. Transversus abdominis release (TAR) is performed as required, depending on hernia size and approximation of the posterior flap. After obtaining adequate dissection of the retromuscular space, the anterior fascial defect is closed, when possible, with an absorbable barbed suture. The mesh is placed in the retromuscular plane, and the posterior flap is closed using an absorbable barbed suture.

### 2.5. Statistical Analysis

A one-to-one propensity score matching (PSM) analysis was conducted to reduce potential bias and to attain comparable groups (rTA-RM and l-IPOM). A caliper of 0.50 of the standard deviation of the logit of the propensity score was used to obtain similar groups regarding the set of covariates. Statistical assessments were performed using SPSS software pack (Statistical Package for Social Sciences for Windows version 29 software). A *p* value of < 0.05 was considered statistically significant.

## 3. Results

After propensity-score matching, 30 patients were included in each group. The standardized differences in pre- and post-matching are shown in Table 1. In both unmatched and matched groups, variables such as age; sex; BMI; ASA score; and risk factors like diabetes, COPD, smoking, and history of wound infection were comparable, with no statistically significant differences. Operatively, l-IPOM had a significantly shorter mean operative time (74.4 vs. 93.2 min, *p* = 0.004); the type of mesh used varied significantly between the two techniques. In the rTA-RM group, Polymesh™ (Betatech Medical, Istanbul, Turkey) (36.6%) and ProGrip™ (MEDTRONIC, Dublin, Irland) (63.3%) were used exclusively, whereas the l-IPOM group utilized a variety of meshes, including Parietex™ (Covidien, New Haven, CT, USA), Synecor™(GORE, Newark, DE, USA), and Symbotex™(MEDTRONIC, Dublin, Irland). Additionally, the use of fixation techniques differed; tacks were used exclusively in the l-IPOM group, while the rTA-RM group utilized surgical glue in 30% of cases, indicating different approaches to mesh fixation (Table 2). Postoperatively, rTA-RM resulted in shorter median hospital stays (1 day vs. 2 days, *p* = 0.003) and lower median VAS pain scores (3 vs 5, *p* = 0.004). Complication rates were generally similar between the two groups, with no statistically significant differences. Neither group had severe complications (Clavien-Dindo Grade III or IV) nor required reoperation. Time to return to work was lower in the rTA-RM group (12.8 vs. 15.2). Postoperative data are shown in Table 3. Total costs were not significantly different (6862 for rTA-RM vs. EUR 6575 for l-IPOM, *p* = 0.32). However, rTA-RM had significantly lower costs for disposable supplies (EUR 1057 vs. EUR 2006, *p* < 0.01), particularly mesh costs (EUR 160 vs. EUR 863, *p* < 0.01). The robotic approach added EUR 1804.11 per case (Table 4).

## 4. Discussion

L-IPOM repair is a technique widely accepted by the scientific community thanks to the ease in treating ventral hernias through the positioning of an intraperitoneal mesh, deriving all the advantages of minimally invasive surgery in terms of recovery [3]. However, faced with complex ventral and incisional hernias and the need to perform component separation, many surgeons have retraced their steps by preferentially using an open approach. Robotic surgery is the latest advance in minimally invasive hernia repair, combining the advantages of open repair with complete abdominal wall reconstruction and restoration of functional anatomy with the wound morbidity and decreased recovery time of laparoscopy [9].

During our experience, the first robotic cases of ventral hernia repair had been addressed with the same approach as laparoscopy (IPOM), subsequently managing to carry out hernia repairs with an effective retromuscular approach; the robotic platform has therefore permitted us to modify our surgical approach, allowing us to perform reconstructions of the abdominal wall with excellent postoperative results.

While robotic surgery offers potential benefits in terms of precision and minimally invasive approaches, its higher costs remain a significant concern in general surgery. The challenge lies in balancing these costs against potential benefits in patient outcomes and long-term healthcare savings. Costs, therefore, represent a limit in the use of robotic platforms, especially in certain procedures such as the repair of abdominal hernias: while robotic ventral hernia repair can offer clinical benefits, these advantages must be weighed against the financial constraints imposed by low DRG (Diagnosis-Related Group) classifications [7].

The nationwide Danish study by Jensen et al. comparing laparoscopic intraperitoneal onlay mesh (IPOM) repair to robot-assisted retromuscular repair for small to medium ventral hernias provides valuable insights into the short-term outcomes of these two approaches. The findings suggest several potential advantages of the robot-assisted retromuscular technique: reduced length of stay and lower readmission rate. While not directly measured in this study, the authors speculated that reduced postoperative pain may explain the shorter hospital stays and lower readmission rates with robotic repair. This aligns with other studies suggesting less pain with retromuscular mesh placement compared to IPOM. They concluded that potentially shorter hospital stays with robotic repair could offset some of the higher equipment costs [10].

Petro et al. provided important evidence comparing robotic and laparoscopic approaches for ventral hernia repair with intraperitoneal mesh. The key finding was that there were no significant differences between the two techniques in terms of postoperative pain, quality of life, complications, or length of hospital stay; this study challenged the rapid adoption of robotic ventral hernia repair by demonstrating no clear clinical benefits over laparoscopic repair despite increased costs. It highlighted the need for robust evidence to guide the implementation of new surgical technologies and techniques [11].

The study by Christoffersen et al. comparing robotic-assisted retrorectus repair to laparoscopic intraperitoneal onlay mesh repair for small to medium-sized ventral hernias raised several interesting points for discussion: the significantly reduced need for local blocks or epidural analgesia in the robotic group suggests this technique may offer superior pain control, and the shorter hospital stay is noteworthy. While not directly addressed in their study, the cost-effectiveness of robotic surgery is an important consideration. The initial high cost of robotic systems needs to be weighed against potential savings from shorter hospital stays, less postoperative pain management, and the use of less expensive uncoated mesh products [12].

In a cost analysis of robotic treatment for large incisional hernias, Mongelli et al. showed that robotic surgery had higher operatory theatre-related costs; however, they were fully compensated by shorter hospital stays, resulting in similar total costs [13].

The findings of our study challenge the conventional wisdom that robotic surgery is significantly more expensive than laparoscopic approaches. Despite the high initial investment and per-case costs associated with robotic systems, the total costs of rTA-RM were comparable to those of l-IPOM placement. This cost parity is noteworthy and warrants further discussion; while the total costs were similar, their distribution differed: the robotic approach incurred an additional cost per case for the use of the robot. However, this was offset by lower costs in other areas, particularly in disposable supplies. This suggests that the higher capital costs of robotic systems might be partially mitigated by savings in other areas. A significant portion of the cost difference in disposables came from mesh expenses. This highlights how technique-specific factors, rather than the use of robotics itself, can significantly influence overall costs. The robotic approach took longer, which typically increases costs; however, this did not translate to higher overall costs, suggesting potential efficiencies in other aspects of the procedure. The shorter length of hospital stay and lower pain scores for rTA-RM patients could contribute to cost savings, although this study did not quantify long-term outcomes.

As surgeons become more proficient with robotic techniques and as case volumes increase, we might expect to see further improvements in efficiency and potential cost reductions. Another interesting result is that regarding the time to return to work, which was shorter in the robotic group, even if it does not directly affect hospital costs, confirmation of these data can have significant implications on the national socio-economic impact, justifying the use of the robotic platform [14]. While not addressed in this study, potential differences in long-term outcomes or recurrence rates could impact the overall cost-effectiveness of each approach.

## 5. Conclusions

This study challenges the assumption that robotic surgery is always more expensive than laparoscopic approaches. It suggests that when considering the total cost of care, robotic techniques can be cost-competitive in certain procedures. However, the higher capital costs of robotic systems remain a significant factor, especially for lower-volume centers.

Future research should focus on long-term outcomes, indirect costs, and broader economic impacts to provide a more comprehensive understanding of the cost-effectiveness of robotic versus laparoscopic approaches. Additionally, as robotic technology continues to evolve and potentially become more affordable, ongoing cost analyses will be crucial to inform healthcare decision making and resource allocation.

## Figures and Tables

**Table 1:** Comparison of study groups in terms of pre-operative variables before and after matching.

	Unmatched Comparisons			Matched Comparisons		
	rTA-RM *n* = 41	l-IPOM *n* = 58	*p*	rTA-RM *n* = 30	l-IPOM *n* = 30	*p*
Age (years), mean ± SD	57.8 ± 13.1	56.6 ± 14.7	0.530	56.6 ± 13.2	57 ± 14.5	0.875
Sex, female, *n* (%)	60 (54.1)	46 (44.7)	0.175	43 (52.4)	40 (48.8)	0.755
BMI (kg/m^2^), mean ± SD	32.6 ± 6.5	31.3 ± 6.7	0.161	32.2 ± 6.6	31.8 ± 7.2	0.564
ASA score, median (IQR)	3 (2–3)	3 (2–3)	0.147	3 (2–3)	2 (2–3)	0.122
Risk factors						
DM, yes, *n* (%)	9 (24.3)	10 (17.5)	0.243	6 (20.7)	5 (18.3)	0.844
COPD, yes, *n* (%)	4 (10.8)	5 (8.7)	0.652	3 (11)	3 (11)	1.000
Smoking, yes, *n* (%)	9 (24.3)	11 (19.4)	0.413	8 (26.8)	6 (20.7)	0.463
History of wound infection, yes, *n* (%)	8 (21.6)	10 (17.5)	0.493	6 (20.7)	5 (18.3)	0.844
Hernia etiology						
Primary ventral, *n* (%)	11 (25.2)	30 (51.5)	0.048	10 (34.1)	11 (39)	0.331
Incisional, *n* (%)	30 (74.8)	28 (48.5)	<0.001	20 (65.9)	19 (61)	0.627
Recurrent hernia, *n* (%)	14 (35.1)	18 (32)	0.666	12 (42.7)	12 (40.2)	0.874

**Table 2:** Operative technical details.

	rTA-RM *n* = 30	l-IPOM *n* = 30	*p* Value
Operative time (min), mean ± SD	93.2 (34.3)	74.4 (32.2)	0.004
Type of mesh, *n* (%)			
Parietex™ (Covidien, New Haven, CT, USA)	0 (0)	8 (26.6)	
Synecor™ (GORE, Newark, DE, USA)	0 (0)	16 (53.3)	
Symbotex™ (MEDTRONIC, Dublin, Irland)	0 (0)	6 (20.0)	
Polymesh™ (Betatech Medical, Istanbul, Turkey)	11 (36.6)	0 (0)	
ProGrip™ (MEDTRONIC, Dublin, Irland)	19 (63.3)	0 (0)	
Use of tacks, *n* (%)	0 (0)	30 (100)	
Surgical glue, *n* (%)	9 (30.0)	12 (40.0)	
Conversion to open surgery, *n* (%)	0 (0)	0 (0)	

**Table 3:** Outcomes and severity of complications according to the Clavien–Dindo classification.

	rTA-RM *n* = 30	l-IPOM *n* = 30	*p* Value
Length of hospital stay, median (range)	1 (0–2)	2 (0–3)	0.003
Median VAS, median (range)	3 (1–5)	5 (2–6)	0.004
Postoperative ileus, *n* (%)	0 (0)	1 (3.3)	0.25
Superficial surgical site infection, *n* (%)	1 (3.3)	1 (3.3)	1.00
Seroma, *n* (%)	3 (10.0)	2 (6.6)	0.93
Hematoma, *n* (%)	1 (3.3)	3 (10.0)	0.25
Clavien-Dindo grade *n* (%)			
Mild complications: Clavien–Dindo Grade I or II	5 (18.1)	7 (23.2)	0.14
Mild complications: Clavien–Dindo Grade III or IV	0 (0)	0 (0)	
Reoperation due to complication, *n* (%)	0 (0)	0 (0)	
30-day readmission, *n* (%)	0 (0)	0 (0)	
Mean time to return to work (days), mean ± SD	12.8 ± 10.2	15.2 ± 13.4	0.13

**Table 4:** Cost calculations of rTA-RM versus l-IPOM ventral hernia repair.

	rTA-RM *n* = 30	l-IPOM *n* = 30	*p* Value
Total cost ^a^	6862 ± 3049	6575 ± 2698	0.32
Hospital cost	3818 ± 642	3723 ± 717	0.8
Total cost of disposable supplies	1057 ± 565	2006 ± 1298	<0.01
Mesh cost	160 ± 92	863 ± 372	<0.01
Mesh fixation cost ^b^	172 ± 83	307 ± 96	0.06
Access instruments cost ^c^	171 ± 64	258 ± 77	0.06
Other supplies cost	454 ± 281	479 ± 252	0.7
Total added cost of the robot per case	1804.11		

^a^ Cost data are presented in Euros as mean ± SD. ^b^ Tackers, sutures, glue sealant, and applicators. ^c^ Trocars, needles, insufflation systems, suture passers.

## Data Availability

The data that support the findings of this study are available from the corresponding author upon reasonable request.
